# “Omics” in Human Colostrum and Mature Milk: Looking to Old Data with New Eyes

**DOI:** 10.3390/nu9080843

**Published:** 2017-08-07

**Authors:** Flaminia Bardanzellu, Vassilios Fanos, Alessandra Reali

**Affiliations:** Neonatal Intensive Care Unit, Neonatal Pathology and Neonatal Section, AOU and University of Cagliari, 09124 Cagliari, Italy; vafanos@tiscali.it (V.F.); re.ale1@virgilio.it (A.R.)

**Keywords:** omics technologies, human colostrum, transcriptomics, proteomics, metabolomics, microbiomics, growth factors, pasteurization, preterm newborns, neonatal intensive unit care

## Abstract

Human Milk (HM) is the best source for newborn nutrition until at least six months; it exerts anti-inflammatory and anti-infective functions, promotes immune system formation and supports organ development. Breastfeeding could also protect from obesity, diabetes and cardiovascular disease. Furthermore, human colostrum (HC) presents a peculiar role in newborn support as a protective effect against allergic and chronic diseases, in addition to long-term metabolic benefits. In this review, we discuss the recent literature regarding “omics” technologies and growth factors (GF) in HC and the effects of pasteurization on its composition. Our aim was to provide new evidence in terms of transcriptomics, proteomics, metabolomics, and microbiomics, also in relation to maternal metabolic diseases and/or fetal anomalies and to underline the functions of GF. Since HC results are so precious, particularly for the vulnerable pre-terms category, we also discuss the importance of HM pasteurization to ensure donated HC even to neonates whose mothers are unable to provide. To the best of our knowledge, this is the first review analyzing in detail the molecular pattern, microbiota, bioactive factors, and dynamic profile of HC, finding clinical correlations of such mediators with their possible in vivo effects and with the consequent impact on neonatal outcomes.

## 1. Introduction

Human Breast Milk (HM) is a complex biological fluid which is considered the best source for infant nutrition, until at least the first six months of life [1,2,3,4]; it contains proteins, lipids, sugars, immune cells and bioactive molecules providing nutrition and also exerting anti-inflammatory and anti-infective functions; moreover, HM also promotes the formation of a correct immune system and supports organ development [5,6,7,8].

HM chemical composition is influenced by many factors such as genetics, geographical origins and environment, lactation stage, alimentation, and maternal nutritional status [9]. 

HM antimicrobial and immunomodulatory factors can give passive immunity to breastfed neonates [7,8,10]. In colostrum, there are higher levels of immunoglobulins (Ig) instead of mature milk (MM), because of their passage between the interstitial spaces of mammary epithelium, whose junctions show progressive increase in tightness during milk maturation [7,11]; higher levels of cytokines and immune cells have also been demonstrated [2,12,13].

It is known that breastfeeding can protect from the onset of obesity, diabetes and cardiovascular disease. Furthermore, human colostrum (HC) seems to have a peculiar role in newborn support and plays a protective role against allergic and chronic diseases, in addition to long-term benefits such as a reduction in systo-diastolic pressure and total cholesterol, decreased risk of Type 2 Diabetes Mellitus (DM2), overweight and obesity [14,15,16,17,18,19,20].

In fact, it seems that HC could contain different products on the basis of the newborn’s characteristics; for example, the HC of preterm neonates shows specific factors to sustain correct growth and maturation, and to prevent specific complications [21].

In this review, we analyzed and discussed the very recent published literature in the English language, found in MEDLINE using omics, transcriptomics, proteomics, metabolomics, microbiomics, pasteurization and growth factors, HC, and preterm newborns as key words. 

Our aim was to provide new evidence in terms of “omics” technologies in HC. We also underlined the presence and the functions of growth factors (GF) in HC; moreover, the importance of HM pasteurization and the role of new pasteurization available techniques is discussed in the last part of this article. 

To the best of our knowledge, this is the first review showing these features and analyzing in detail the molecular pattern, microbiota, bioactive factors and dynamic profile of HM during maturation stages, finding clinical correlations of such mediators with their possible in vivo effects and the consequential impact on neonatal outcomes.

Casado et al. published on 2009 a review analyzing the results of some “omics” studies which considered the macronutrient composition (proteins, oligosaccharides and lipids) of human and bovine mature milk and colostrum. This paper pointed out the advantages of using a “omics” approach in milk studies and its importance for future perspectives [22]. 

## 2. Transcriptomics 

The analysis of the genes selectively expressed by a cell, providing information on the specific function of that cell depending on the phase in which it is found, helps in understanding its role and contribution. In mammary cells, the activated genes are different according to the lactation stage to support milk production or the involution of the gland.

This evaluation can be conducted in a non-invasive way through the analysis of RNA obtained from milk cells to detect specific gene expression of mammary epithelial cell (MEC) exfoliated, directly reaching and potentially influencing the intestinal mucosa of breastfeed neonate [23].

Sharp et al. [23] first analyzed HM cell trascriptome modifications during various lactation phases. As a result, although on a small number of samples, they described very interesting findings: during the first lactation phases, genes responsible of milk production and tissue development were the most expressed (such as carboxyl ester lypase (*CEL*), which promotes lipid digestion; oleoyl-ACP hydrolase (*OLAH*), which takes part in fatty acid byosinthesys; the folate receptor 1 (*FOLR1*) to regulate milk protein synthesis; butytrophilin (*BTN1A1*), which plays a role in milk lipid secretion; arginase II (*ARG2*), which is involved in proline biosynthesis; parathyroid hormone like-hormone (*PTHLH*) regulating calcium levels in milk; carbonic anydrase 6 (*CA6*) previously found in salivary glands; saliva and mammary lactating glands involved in the hydration of carbon dioxide; and mucin15 (*MUC15*) cell surfaces associated protein mostly expressed in human placenta [24], alpha-lactalbumin and casein genes). Moreover, the Signal Transducer and Activator of Transcription genes (*STAT*) have also shown a role in mammary cells proliferation and milk formation; *STAT3* and *STAT5A* resulted upregulated during lactation, *STAT1* resulted downregulated while *STAT5B* and *STAT6* did not show fluctuations [23].

*STAT6* seems to be involved in mammary gland development and milk production [23,25]; and *STAT5* is involved in milk production in response to prolactine levels [23,26,27]. On the contrary, the increase in *STAT3* expression during lactation was not predictable, since it is known for its role in apoptosis and involution of the mammary gland; in fact, it seems to be downregulated in mice, suggesting different roles of *STAT* genes in lactation control among different species [23,28]. 

In the same study, during the peak of milk production, 1178 genes were overexpressed and 1318 downregulated instead of the prepartum phase. Among these, genes regulating cell movement, maintenance, interaction and junctions or immunitary response increased. Otherwise, during involution, the most expressed genes were *STAT3*, *NF-KB*, *IRF-5*, *IRF7*, with downregulation of synthesis factors; as a result, in this phase 1398 genes were upregulated while 763 showed lower expression instead of a milk production phase. A very interesting finding was that only eight genes were differentially expressed when milk of the involution phase versus HC was compared [23] and, in our opinion, these could represent the genes involved in HM synthesis and production.

Moreover, during mastitis, genes sustaining milk production were expressed in conjunction with anti-inflammatory, immunitary, and defense related factors; 248 genes were upregulated, including *STAT1*, while 961 genes were downregulated versus healthy HM [23].

## 3. Proteomics

In HM, proteins constitute a fraction of about 3% [1,29], mostly composed of whey proteins and casein. HC contains higher levels of proteins and a higher proportion of whey instead of mature milk (MM) [30].

HM peptides show different functions, such as immunomodulatory, antibacterial, antioxidant, antithrombotic, mineral binding, antihypertensive activity, and opioid antagonism effect [21,31,32,33,34]. 

HM from mothers of pre-terms contain higher concentrations of total proteins, but also fat, carbohydrates [35,36] and free amino acids, lower levels of calcium [37] and also differs in proteome when compared with HM of full term neonates [35,38]. 

Several studies have reported that a different protein expression can influence both the composition and biological functions of HM, but little is still known on this topic [1].

In the following sections, we report the results of the most recent studies analyzing the characteristics and variability of HM proteins and peptides in colostrum, especially in gestational diabetes mellitus (GDM), intrauterine growth restriction (IUGR) and macrosomic fetuses. Moreover, the role of cytokines and chemokines and variability of Milk Fat Globules Membrane proteins (MFGMs) will be considered.

### 3.1. New Evidence in Proteomics

Whey proteins in HC, bovine colostrum (BC) and MM, have been evaluated by Yang et al. [1] in a recent study where the authors described the upregulation of 297 proteins in HC, mainly related to immune system processes. Among these, the most expressed were the Ig kappa chain V-III region IARC/BL41, Ig kappa chain V-I region EU, chitinase-3-like protein 2, V5-6 protein, serum amyloid A protein (which presents cytokine-like activities) and myosin-reactive immunoglobulin heavy chain variable region [1], zinc-alpha-2-glycoprotein, clusterin, neutrophil defensin 1, immunoglobulin J chain, cDNA FLJ90170 fis, IGH@ protein, full-length cDNA of neuroblastoma, putative uncharacterized protein and IGL@ protein. This differential protein expression underlines the variability of HC immunological features [1].

Many differently expressed whey proteins in HC are also involved in enzyme regulatory activity, modulating several biological functions; for example, high levels of thyroxine-binding globulin have been detected [1,39]. Moreover, many proteins also participate in complement and coagulation cascades, phagosome related processes, hematopoietic cell lineage, PI3K-Akt signaling pathway, protein processing, glycolysis and gluconeogenesis, lysosomial processes, the biosynthesis of amino acids, NF-kappa B signaling pathway, antigen processing and presentation, and intestinal IgA production [1,40,41].

Proteomic variability has also been described by evaluating milk-derived exosome proteins of HM and bovine milk (BM) [42]. Exosomes, membranous microvescicles secreted by several cells and found in many biological fluids including milk [42,43,44], are involved in important functions such as intracellular and extracellular communication and immunity and newborn immune system development; moreover, since they contain mRNA and microRNA, they could also represent potential disease biomarkers [45,46,47]. 

The results of this analysis confirmed a large variety and complexity of exosome-derived proteins and the great variability between human and bovine samples. In HC, plastin-2, Ig gamma-1 chain C region, Ig kappa chain V-III region, lysosome-associated membrane glycoprotein 1, and peroxiredoxin-1 showed higher levels. Plastin-2 is involved in phosphorylation and leukocyte function, acting as a potential biological marker and showing antibacterial activity [42,48,49,50]. 

The highest expression variability has been detected in proteins associated with ribosomes, regulation of actin cytoskeleton (potentially modifying signal transduction and actin related cellular functions) [51], glycolysis, gluconeogenesis, leukocyte transendothelial migration, aminoacyltRNA biosynthesis, pentose phosphate pathway, galactose metabolism and fatty acid biosynthesis.

Moreover, high levels of Ig kappa chain V-III region CLL and anti-thyroglobulin heavy chain variable region have been detected in HC [42].

Many differentially expressed proteins are involved in the modulation of newborn immune response and protection against oxidative stress; among these, 15 were highly expressed in HC such as neutrophil defensin 1, immunoglobulin J chain, cDNA FLJ14473 fis, IGH@ protein and superoxide dismutase [42,52]. In addition, 17 exosomes proteins upregulated in HC were involved in transport mechanisms, potentially playing important roles in signal transmission; additionally, the solute carrier family 2 and ATP synthase subunit delta was overexpressed.

In conclusion, a great variability has been detected in both human and bovine samples, with a higher number of differentially expressed immune-related proteins in HC [1].

### 3.2. Cytokines and Chemokines

The presence of several cytokines in HM, showing a higher concentration in HC, has already been reported in many studies. Furthermore, interest in cytokines and chemokines in HC content is increasing, especially to clarify which maternal or pregnancy-related factors can influence their concentrations [2,53,54,55,56,57]. The very recent study of Zambruni et al. [2], even if conducted on a peculiar population of mothers (*n* = 223 Peruvian mothers living in resource-limited conditions and giving birth to low birth weight neonates (LBW)), pointed out that the course of pregnancy showed a great influence on HM composition at birth and during the first weeks of lactation, potentially influencing infant mucosal immunomodulation and clinical outcomes. In particular, 13 cytokines and chemokines related to innate immunity were highly represented in HC instead of in mature milk (MM) [2]. Lower levels of pro-inflammatory cytokines in HC have been associated with peripartum infections, spontaneous preterm delivery and VLBW neonates, especially those related to the immune system, which could protect the newborn against the mucosal invasion of pathogens. In contrast, high levels of these mediators are secreted by mothers affected by hypertension. Moreover, Cesarean section, twin pregnancy or antenatal steroids showed only a minimal impact on HC cytokines content [2].

Interestingly, the transmission of cytokines from mother to neonate could represent a key mechanism of the breastfeeding protective effect against necrotizing enterocolitis (NEC) and sepsis [1,58,59].

### 3.3. Gestational Diabetes Mellitus 

Gestational Diabetes Mellitus (GDM) determines an increase in pregnancy-related morbidity, leading to a higher risk of maternal infections, eclampsia and premature delivery; in addition, it can also induce fetal macrosomia. Moreover, as a long-term effect, GDM is predisposed to metabolic and cardiovascular disorders. Little is known about the effects of GDM on HC composition [60].

Several studies have shown a correlation between maternal insulin sensitivity and immunological HC status, demonstrated a lower level of IgA, IgG, and C3 proteins in HC of hyperglycemic mothers [7,61,62] and lower levels of IgA and its glycosylate form in the transitional milk (TM) of GDM mothers [63].

It has also been demonstrated that GDM greatly influences HM glycome [63] and Grapov et al. [7] described different protein profiles in the HC of mothers affected by GDM (*n* = 6) compared with healthy controls (*n* = 12). In this study, 27 proteins resulted in predictors of GDM; among these, 10 showed a statistical significance between the two categories. The HC of GDM mothers presented higher levels of apolipoprotein D, Ig heavy chain V-II region ARH-77, and prostasin. Apolipoprotein D level usually decreases during the postnatal period in breastfeeding women; similarly, there is a decrease in Ig levels, including Ig heavy chain V-II region ARH-77, due to tight junction closures. In contrast, these mediators increased in HC from GDM mothers, and this observation seems to support the hypothesis that GDM could determine a delayed lactogenesis previously demonstrated for obese women [11]; moreover, GDM could also interfere with the occurrence of copious lactation [7].

Lower levels of alpha-2-HS-glycoprotein, apolipoprotein A1 and E, 14-3-3 protein zeta/delta, protein disulfide-isomerase, protein DJ-1 and protein FAM3B (pancreatic derived factor) have also been detected in the HC of GDM mothers. In the same study, Grapov et al. [7] demonstrated lower levels of lipid synthesis proteins in the HC of GDM mothers and found lower lipid levels in GDM mothers’ MM. This result had been previously described by Morceli et al. [60] and appeared to depend only on GDM and hyperglycemia and not on maternal pre-pregnancy BMI [7].

Basaran et al. [64], studying pregnancy hyperlipoproteinemia, showed lower plasma levels of LDL and HDL in GDM women [64].

Finally, it emerges that a specific group of proteins is reduced in HC, such as alpha-2-HS-glycoprotein (involved in metabolic response against stress), apolipoprotein A1 and E (responsible of VLDL and LAD transport), FAM3B protein (able to limit vascular damage), and proteins involved in lipid synthesis [7]. The reduced expression of this group of proteins, if confirmed by further studies, seems to point out a long-term protective role of GDM mother’s HC on newborns. 

Several studies have investigated the biochemical and immunological changes of HC related to maternal overweight and obesity. It is known that this condition is associated with low grade inflammation, confirmed by an increase in serum cytokines and inflammatory markers in maternal and fetal blood [14,17,65,66] and can be potentially aggravated during pregnancy, representing a disease predisposing factor both for the mother and also for the fetus (and later in life) [14,67,68,69].

Fujimori et al. [14] evaluated modifications of hormonal and immunological components comparing HC samples from normoweight (*n* = 15), overweight (*n* = 15), and obese mothers (*n* = 15), demonstrating an increase of adiponectin and leptin in obese mothers; globally, these results suggest that HC can minimize inflammation and protect against negative fetal programming, which could lead to obesity later in life [14].

However, there are still dissenting opinions about the high levels of adiponectin concentration in HM; for example, some authors had previously associated it to lower weight gain at 6 months of life or to childhood obesity [70,71].

In the literature review, we did not find correlations between proteome, transcription factors and metabolites in HC from mothers delivering macrosomic fetuses compared with mothers affected by GDM, but it could result in interesting and relevant research in the search for such analogies in these populations. 

### 3.4. Macrosomic Newborns

In macrosomic newborns, short-term risk of metabolic problems, electrolyte disorders, glycopenia and hyperbilirubinemia have been described in References [21,72]. In addition, these babies have also shown long term problems such as metabolic disorders, cardiovascular disease and cancers [21,73,74,75]. 

Cui et al. [21] demonstrated a different composition between the HC of mothers delivering macrosomic newborns (*n* = 6) when compared with mothers of term non macrosomic newborns (*n* = 6) through evaluating the endogenous peptides of both categories [21]. First, the authors found more than 400 peptides originating from about 34 precursors: among these, 29 showed significantly different expression between the two categories (15 presenting higher levels in HC from mothers delivering macrosomic newborns). Among the identified peptides, 279 were derived from β-casein and the others originated from α-casein (CASA), k-casein (CSN3) and other precursors [21].

In the same study, Cui et al. [21] proposed a casein 24 (α-casein derived) antimicrobic activity against *E. coli*, *Y. enterocolitica* and *S. AUREUS*, frequent species in neonatal intensive care units (NICU). This could be useful, as it is well known that the widespread use of antibiotics carries a high rate of resistance. Casein 89 (k-casein derived and highly expressed in HC from mothers delivering macrosomic newborns) has shown an inhibitory effect on preadipocyte proliferation, protecting from successive weight gain. Thus, the different composition demonstrated in HC samples from mothers of macrosomic newborns could protect from obesity, diabetes, or cardiovascular disease onset [21,76,77,78]. Further studies are needed to confirm and clarify these findings on more numerous samples.

### 3.5. Milk Fat Globules Encircled by a Membrane 

In HM, fat is secreted as globules (Milk Fat Globules, MFGs) encircled by a membrane (MFGM) of lipids and proteins [79,80,81,82]. MFGM proteins constitute a small percentage of HM proteins, representing about 1–2% of the whole protein content, but plays several roles [83,84]; for example, MFGM act as transporters, emulsifiers, and stabilizers of lipids [79,81,85,86], and can also show anti-infective effects [79,87,88]. 

Moreover, some MFGM proteins take part in cell death, adhesion or locomotion, signaling pathways, response to stimuli, localization, protein or nucleotide binding, enzyme inhibition, antigen presentation, carboxylic acid or amine binding action, cell junction, complement and coagulation mechanisms, and leukocyte migration [79].

Our knowledge of MFGM functions and composition has largely increased in the past few years [22]. In HM and BM, a large number and a great variety of MFGM has been detected during different lactation stages, with a different expression in the two kinds of samples. In particular, the IgG H chain, Ig heavy chain variable region, neutrophil defensin 1, lactadherin, immunoglobulin lambda-like polypeptide 5, and myosin-reactive immunoglobulin heavy chain were higher in HC [79].

The most represented MFGM in HC are histone H3 (contained in chromatin) [79,89] and lactoferrin (playing a role in the immune system and showing antibacterial, antiviral, anticancer, antiparasitic, catalytic, antiallergic and radioprotective functions) [79,90]. 

Yang et al. also described five caseins in HM MFGM fractions, such as α-s1-casein, α-s2-casein, β-casein, k-casein, which should provide amino acids, calcium and phosphate for newborns [79,91]. 

The IgG H chain, Ig heavy chain variable region, polymeric Ig receptor and Immunoglobulin J chain have been detected in great quantity in HC. In conclusion, MFGM upregulated in HC plays an important role in newborn immunity system development and, furthermore, the high level of clusterine may be beneficial for nutrition and protection against oxidative stress and aging [79,92]. 

## 4. Metabolomics

Many studies have been performed to evaluate the macronutrient composition of HM; however, micronutrients have been poorly investigated despite representing significant and relevant metabolites [35,36,37].

Metabolomic studies have been performed both on HM [5,37,93,94,95,96,97,98] and BM [35,99,100,101,102] that have also focused on metabolite changes during lactation [37]. HM metabolome, evaluated with nuclear magnetic resonance spectroscopy (NMR), has shown differences according to Gestational Age (GA), lactation phase [35], exposure to exogenous substances and it is different from BM and formula milk (FM). Proteins [36], lipids [103], lactose [104] and other mediators vary according to GA and lactation time [35] as the HM metabolome can change according to the neonates’ necessities.

Cesare Marincola et al. [95] conducted the first study analyzing the HM metabolome of the milk from mothers of pre-term versus full term neonates and compared this to FM [95]. Since this article, several other studies have evaluated the HM metabolome with the most relevant findings described in a recent paper by Fanos et al. [105].

The different metabolic profile of preterm HM compared with full term HM during three periods of lactation (HC, transitional milk (TM) and MM) was demonstrated by Sundekilde et al. [35] on *n* = 92 total samples from *n* = 45 mothers. Furthermore, Spevacek et al. [37] demonstrated a higher variability in preterm samples and identified and measured 69 metabolites at three time points (HC, TM and MM); in particular, 15 sugars, 23 amino acids, 11 energy-related metabolites, 10 fatty acids (FAs), 3 nucleotides, 2 vitamins and 5 bacteria-associated metabolites, and also showed that lacto-*N*-tetraose and lysine decreased during term milk maturation without significant changes in preterm samples [37].

Sundekilde et al. [35] detected changes in carnitine, caprylate, caprate, pantothenate, urea, lactose, oligosaccharides, citrate, phosphocoline, choline, and formate during milk maturation, underlying that preterm HM metabolome varied during 5–7 weeks postpartum, probably reaching the composition of term milk after this time and independently from premature GA. In the same study, higher levels of valine, leucine, betaine, creatinine, pantothenate, citric, and lactic acid in HC and TM were demonstrated [35].

In preterm HC, there was an increased level of oligosaccharides (HMOs), citrate, and creatinine. Higher levels of fucosylated oligosaccharides, fucose, *N*-acetylneuramic acid, and *N*-acetylglucosamine were detected in HC than in MM. Beta-hydroxybutyrate levels did not show variations according to milk stage in full term samples. Citrate, lactose, phosphocoline, Fucosyl moieties, *N*-acetylneuraminic acid, *N*-acetylglucosamine, 3′-siallylactose, 6′-siallylactose, lacto-*N*-difucohexaose I (LNDFH I), glutamate, choline, and formic acid had higher in preterm samples [35].

Villasenor et al. [106] demonstrated a different composition of term HC samples during the first lactation week compared with samples of the fourth lactation week, with higher levels of lysolipids, phospholipids, alpha-tocoferol, cholesterol, fucose, furanose, d-glucosaminic acid, and lower levels of oleic, linoleic, palmitoleic, gluconic and idrossiadipic acids, di- and tri-glycerids and lisophosphatidyletanolamine in HC [106].

Our research group, in collaboration with Turin University, conducted a comparative study between two subcategories of premature milk, underlining the differences existing in their metabolome. In fact, HC, TM and MM were compared in extreme preterms (23–28 weeks of GA) and moderate preterms (32–34 weeks), finding more evident sample separation in HC and TM instead of MM [107].

### 4.1. Carbohydrates 

HM metabolome also varies according to maternal phenotype; in HM oligosaccharides (HMOs), secretion depends on both blood group and genic expression of glicosyltransferase family enzymes, such as alpha-1-2-fucosyltransferase FUT2 (codified by *gene Se* and determining secretor or non-secretor status) and alpha-1-3-4-fucosyltransferase FUT3 (codified by *gene Le* and expressing positivity or negativity for the Lewis Group) [108].

According to these findings, maternal phenotype can be classified as follows: Se+/Le+ (Le positive, secretor), Se+/Le− (Le negative, secretor), Se−/Le+ (Le positive, non-secretor) and Se−/Le− (Le negative, non-secretor). Se+ milk was higher in 2-fucosyllactose (2-FL), lacto-*N*-fucopentaose I (LNFP I), and other alpha1-2 fucosylated olygosaccarides resulting in protection against intestinal dysbiosis. In the study of Spevacek et al. [37], out of 45 mothers, 28.9% resulted in non-secretors, and differences in fucosilated HMOs also depended on this condition. In milk from non-secretors, oligosaccharides, α1,2 fucosylated structures have not been detected, while higher levels of 3′ fucosyllactose (3′-FL) have been found. 

According to the detection of 2′-FL in NMR spectroscopy, secretors and non-secretors [37,98,109] mothers’ samples were segregated in two different areas of the plot [37].

HMOs and their components decreased with milk maturation [35,106]; moreover, Lacto-*N*-tetraose, LNDFH I, 3′-sialyllactose, 6′-siallylactose, fucose, *N*-acetylglucosamine, and *N*-acetylneuraminic acid had higher results in preterm milk [35,110]. 

According to other authors’ results, Spevacek et al. [37] found that the total HMOs and sialic acid were higher in preterm HM [37,104,111,112] where samples of carbohydrates and HMOs also showed a great variability in concentration [37,110,112]. 

Higher levels of 3′-galactosillactose (3′-GSL), 2-hydroxybutyrate, methionine and acetoin, but not of dimethyl sulfone, have also been found in HC and decreased during lactation [37]. 

In term HM samples, lactose, 3-FL and glucose increased with milk maturation, while 2′-FL, 3′-GSL, 3′-SL, 6′-SL, LNFP III and fucose decreased. LNFP III did not change in preterm samples over time. 3′-FL increased during the first 28 days in both term and preterm groups, while lacto-*N*-fucopentaose III and lacto-*N*-neotetraose were lower in preterm instead of term HC, but were similar at 28 lactation days.

Differences between mature HM samples in the intervals before and after 26 weeks have not been described, suggesting that after the variability detected in HC, the metabolome tended to re-align after several weeks of milk maturation [35]. 

The importance of HMOs was also related to their possible influence on gut microbiota reducing the incidence of NEC [35,113].

### 4.2. Amino Acids and Creatinine

In the analyzed studies, amino acids showed high variability across all lactation phases, especially in HC [37].

In particular, some of these decreased during lactation [35,103]; glutamate increased [35,114] while valine and leucine increased more in preterm samples than in full term HM [35,115,116]. Higher levels of phosphocoline was recently found in preterm samples, in contrast to the results of previous studies [35,117].

The presence of betaine and coline in preterm HC (also demonstrated by Sundenkilde et al. [35]) and the increasing levels of glicerophosphocoline during the first three months of lactation [93] suggest that these metabolites could play an important role [35,93]. Coline and its derivatives contribute to cellular membrane integrity, playing a pivotal role in preterm homeostasis; and also contribute to cerebral maturation and results as a precursor of acetilcoline [118]. Coline and its derivative betaine are involved in the prevention of homocisteine accumulation, reducing the risk of cardiovascular diseases [107,119].

The high levels of alanine in HC, partially converted in glutamate and also involved in energetic metabolism, confirmed that this amino acid is fundamental in HM, as previously demonstrated by Spevacek et al. [37] and Andreas et al. [93].

Sundekilde et al. [35] detected increasing levels of leucine, isoleucine and valine during milk maturation, which is an important finding since their role in cerebral development is well known [120], as well as immunitary system constitution [121], hepatic regeneration [122], and glucose metabolism [123].

Glutamate must be converted in glutamine to pass through the hematoencefalic membrane and reach the brain [124], and different authors have found increasing levels of these two metabolites during milk maturation [5,35,37]. 

Methionine is involved in carnitine synthesis, promoting LC-FAs transport into the mitochondrial matrix where they can be used for energy production; moreover, methionine also shows cardioprotective and vasoprotective effects and improves lipid metabolism [125,126]. We found increased levels of this metabolite in extremely preterm TM samples [107] and Spevacek et al. detected increasing levels during the first month [37].

In our results, HC samples from mothers delivering extremely preterm newborns showed higher levels of alanine, glutamate, glicerophosphocoline, and creatinine, and lower levels of coline, phosphocoline, and pantotenic acid instead of the moderate preterm samples [107].

In term milk, 2-aminobutyrate, alanine, carnitine, glutamate, glutamine, histidine, urea and valine increased during lactation while alanine was higher in the preterm samples. Acetylcarnitine, betaine, lysine, isoleucine and taurine decreased in samples of mother of full term neonates, remaining unchanged in preterm HM samples [37].

Creatinine is involved in energetic metabolism and brain development. It is a phosphocreatine metabolite, which can be found in scheletric and cardiac muscle, brain, liver and kidney. Our group [107] detected high levels of creatinine in the HC and TM of extreme preterms and Sundenkilde et al. [35] also found high levels in the HC of term neonates; creatinine, *N*-acetylaspartate and glutamate had higher results in the first three months of life, according to brain development necessities [127].

In conclusion, creatinine and most of the amino acids showing higher levels in HC and in particular in preterm samples, seemed to improve both energy production and newborn nervous system biosynthesis to sustain neonate development, in particular in the vulnerable category of prematurity.

### 4.3. Fatty Acids (Lipidomics)

HC and MM lipidic composition is about 1.9–2.3% and 3.5–4.5%, respectively, with a high percentage of tryglicerides. Palmitic, oleic, linoleic and alpha-linolenic acids are the most abundant fatty acids (FAs) detected in these samples [128,129,130]. 

Maternal age, nationality, parity, GA, maternal body mass index (BMI) and diet, stage of lactation, GDM, number and duration of daily breastfeeding are all factors that can influence HM lipid content [128,130,131,132,133,134,135,136]. 

The most recent article evaluating these effects, was the study conducted by Sinanoglou et al. [128], which suggested a prominent influence of maternal nationality and age on FA profile when compared to mode of delivery and maternal body mass index (BMI). In particular, in their *n* = 97 HC samples from Greek mothers, maternal nationality mainly influenced the saturated fatty acids (SFAs) and monounsaturated fatty acids (MUFAs) profile; oleic acid, other MUFAs and eicosanoic acid had higher results instead of samples of HC from mothers belonging to other nationalities, probably due to the influence of the high content of olive oil, vegetables, grains and legumes in the Mediterranean diet. As a consequence of such alimentation, HC from Greek mothers was antiatherogenic and antithrombotic, underlining that maternal diet could represent a dietetic strategy to improve newborn health [128].

The influence of GDM on FAs of HC was first evaluated by Chertok et al. [137] on GDM mothers (*n* = 29) who were compared with healthy controls (*n* = 34). As a result, although the study only considered a small number of samples, four essential ω-6 polyunsaturated FAs (ω-6 FAs) such as γ-linolenic, eicosatraenoic, arachidonic and docosatetraenoic acids had higher results in the HC of GDM mothers. The implication of this data must be fully clarified through further studies, but it appears that ω-6 FAs could represent bioactive substances for milk fortification and support infant neurodevelopment. Moreover, ω-6 FAs may constitute an additional metabolic mechanism to compensate for insulin sensitivity damage in the newborns of GDM mothers [137,138]. At least, the authors also underline that the timing of sample collection greatly influenced the metabolites and FA concentration [137].

A correct knowledge of the factors determining FA composition could help to formulate specific dietary regimes for mothers to improve milk nutritional power [128]. 

### 4.4. Metabolomics of Intrauterine Growth Restriction Neonates

In the literature survey, there were only few data on the composition of HC from mothers delivering Intrauterine Growth Restriction (IUGR) neonates. In fact, only three studies have investigated fat content in HC from this group of women, reporting discordant conclusions. A high birth weight (BW) has been correlated with an increase in HM protein and fat content, while the levels of other nutritional components such as carbohydrates, seem not to be related to this factor. Armoni et al. [139] and Lubetzky et al. [140] did not detect significant differences in lipids and FAs in IUGR mothers’ HC. In a recent work published in 2017, an increased concentration of FAs (lauric, tridecanoic and iso-palmitic acids) were reported in HC from mothers delivering neonates with a BW lower than the 20th centile [128,139,140].

A recent metabolomic paper analyzing the effects of breastfeeding during the first week of life, pointed out very interesting data about diet related effects on IUGR metabolism [141]. At birth, IUGR neonates present different metabolic profiles instead of those appropriate for GA (AGA) neonates. Urinary metabolites, such as threonic acid, pseudouridine and ribose were evaluated and suggested an impaired glucose tolerance. Administration of FM did not show any influence on this metabolic pathway, so the urinary metabolites detected in such neonates were the same and indicated an increased carbohydrate synthesis; moreover, the administration of IUGR HC, containing a lower energy level, resulted in lipolysis activation (demonstrated by the presence of aconitic acid, aminomalonic acid, and adipic acid). This study also suggested how a maternal milk diet conducted during the first week of life, similarly influenced IUGR, Large for Gestational Age (LGA) and AGA newborns’ metabolism, instead of the clustering evident at birth. Finally, the authors showed that HC administered to IUGR could correct and re-align their metabolism to AGA neonates [141].

## 5. Microbiomics

The composition and metabolic interaction of microbiota is an epigenetic determinant of human health status [12,142]. Microbial communities have been demonstrated as complex and individual specific [12,143]. Moreover, many microbic-related metabolites constitute a linking ring between metabolomics and microbiomics.

HM is an important source of commensal bacteria for newborns, representing a dynamic ecosystem for several hubs that changes during the lactation phases [144]. Many studies have confirmed the diversity of HM and HC bacteria [145]; in particular, more than 200 species of 50 different genera have been described [144,146]. These represent a source of probiotics with a central role in the first gut colonization of newborns [144,147,148], which ingest about 1 × 10^5^–1 × 10^8^ bacteria every day [143,144,145,146,147,148,149]. These bacteria could be involved in modulating gut tolerance, giving a balanced immune system stimulation and silencing reactions versus some DNA sequences. Moreover, this evidence suggested the importance of bacterial supplementation of FM [12,150,151]. 

HM influences immunity and gut microbiota through its components, especially HMOs, which represent natural prebiotics [144,145,146,147,148]. Lactic acid bacteria found in HC could stimulate immunity, protect against gastrointestinal infections and improve food nutritional properties [144,145,146,147,148,149,150,151,152]. 

It has been demonstrated that the full maturation of HM microbiota occurs at about one month of life [144]. 

Boix-Amoros et al. [143] described *Staphylococci* spp. and *Streptococci* spp. during all lactation phases, although microbiota showed different patterns at three lactation stages, suggesting an evolution during milk maturation (also evidenced by different microbic-related metabolites) [12,143]. Both HC and MM contain lactic acid and anaerobic bacteria, although anaerobic intestinal bacteria are more represented in MM [144].

According to Cabrera-Rubio et al. [153], in HC there was a high prevalence of *Weisella* spp., *Leuconostoc* spp., *Staphylococci* spp., *Streptococci* spp. and *Lactobacilli* spp., while during the period between the first and the sixth month of life there was an increase of *Veillonella* spp., *Leptotrichia* spp. and *Prevotella* spp. species [153]; HC played roles as both a probiotic and prebiotic, containing some HMOs that newborn can digest due to gut *Bifidobacteria* spp., *Lactobacilli* spp. and *Bacteroides* spp., which are able to degrade sugars for energy production. These species progressively increase in neonates during the first months of life [154,155,156]; their proliferation is useful in preventing aggressive pathogen invasions (such as *Salmonella* spp., *Lysteria* spp. and *Campilobacter* spp.) and in the production of short chain fatty acids (SCFA) involved in gut mucosa homeostasis and in lipid metabolism [6,157].

The study of Damaceno et al. [145] analyzed HM samples of healthy women (*n* = 47) evaluating potential probiotic bacteria; the highest bacterial concentration was found in HC (mean 3.9 log10 CFU/mL) and *S. epidermidis* was the predominant species. In their sample, a probiotic role seems to be played by *L. gasseri*, *B. breve* and *S. salivarius*, and these species could represent possible candidates to be used as probiotics in FM [145].

In the HC of Italian mothers, *Abiotrophia* spp. (a variant of *Streptococci* spp. normally found in the oral cavity, gastrointestinal and genitourinary tract), *Actynomicetospora* spp., *Aerococcus* spp., *Alloicoccus* spp. (a member of vaginal microbiota, [147,158]), *Amaricoccus* spp., *Bergeyella* spp., *Citrobacter* spp., *Desulfovibrio* spp., *Dolosigranulum* spp., *Faecalibacterium* spp., *Parasutterella* spp., *Rhodanobacter* spp., and *Rubellimicrobium* spp. have been described as the most represented hubs [144].

It is not currently well known how mothers’ intestinal microorganisms reach the mammary epithelium, but two hypotheses have been proposed: an entero-mammary pathway with vascular translocation (via dendritic or CD18+ cells), and/or a retrograde flow which may occur during nursing [144,146,159]. 

HM bacteria can also protect against several diseases, perhaps reducing respiratory and enteric infections [144,148,160]. 

Furthermore, it is possible that the overgrowth of one bacterial component over the others could lead to dysbiosis and pathologic states (such as in mastitis) [161]. In fact, during mastitic infection, it has been demonstrated that microbiome lose their diversity, with a predominance of bacterial pathogen species [162]. Probiotics seem to be effective in lactational mastitis treatment and they could also play a role in breast cancer prevention [12,163,164,165]. 

Analyzing HC composition, a high variability in relation to maternal factors (diet, lifestyle and obesity) and postnatal factors (such as GA or antibiotic therapies) has been shown. Diet was the most relevant modifier of microbiota composition [144,166]; moreover, it varies in different populations, for example in mothers from Italy and Burundi [144], a mother’s weight and modality of delivery influence HM microbiota composition [145]. 

If compared with other sources of human microbiome, HC bacterial composition shows singular features. Elective caesarean section is a factor which only modifies qualitative bacterial composition of HC, making it more similar to the oral cavity microbiome. In contrast, GA and antibiotic therapy administered to the mother just before delivery, are the two postnatal factors influencing both qualitative and quantitative composition of HC microbiome.

## 6. Other Micronutrients and Metallomics

Among HM micronutrients, an essential role is played by iodine, since it is a constituent of thyroid hormones. Its level is influenced by maternal diet and has mainly been found as iodide. Determining the iodine exact level and physiology in HM is important to enrich FM with the right and safe quantities of this mineral [167].

A growing interest also regards the HM trace elements’ content, such as Fe, Cu, Zn, Se and I, which show a great impact on human health [168]. HM is a great source of Ca and provides Zn and Fe as well as containing traces of Mg, Mn, and Cu [169,170].

In HM, metals are mostly bound to albumin and lactoferrin, but also to the human milk secretory IgA (sIgA) [169].

The iron requirement is higher for preterm neonates; moreover, infants fed with FM can be at risk of iron-deficiency, so both of these categories should receive a supplement for correct growth and development [168]. Although some studies have pointed out a higher or slightly higher iron content in HC instead of MM [169,170,171,172,173] and in preterm samples instead of full term babies’ mothers HM [174,175], the results of Fernandez-Sanchez et al. did not show statistically significant variations in iron content during the first month of lactation, not even in preterm samples [168]. The authors also showed a greater iron availability in HM compared with FM, despite a higher total concentration in the second sample; this could depend on the chemical form Fe^3+^ in HM, which should also be used in the iron-fortified formulas [168].

Although it is known that FM contains more zinc than HM, breastfed neonates present with higher plasmatic levels of this ion [169]. In HM, only about 5% of the total amount of zinc is bound to proteins, while the highest percentage is bound to citrate [176].

Finally, the recent study of Fernandez-Mendez et al. [177] analyzed the Zn content during the first month of lactation, showing high levels of this ion in HC (decreasing over the time) both in preterm and full-term samples [177].

## 7. Growth Factors

HC is rich in growth factors (GF), which are reported in Table 1 in association with their functions. These bioactive components play a crucial role during the processes of adaptation to extrauterine life and act on different organs and tissues (bowel, nervous system, hematopoietic system, vascular system), promoting development and maturation. They also can pass through the gastrointestinal barrier without undergoing digestion processes, reaching target cells in a bioactive form. Higher GF levels have been detected in HC from women delivering premature neonates, with significantly higher levels in mothers of extremely low birth weight (ELBW) newborns [178]. 

### 7.1. Enteral Growth Factors 

The gastrointestinal tract provides both a nutritional and a barrier function, undergoing profound mutations after birth. Its maturation processes are regulated through complex mechanisms by a high number of agents; among these, growth factors (GF) play an important role, reaching the intestine through HM and locally exerting their action. Compared to mature HM, HC contains a higher concentration of GF, such as epidermal growth factor (EGF), hepatocyte growth factor (HGF), insulin-like growth factors (IGFs), transforming growth factor (TGF), fibroblast growth factors (FGF-6 and FGF-7), and granulocyte-colony stimulating factor (G-CSF), promoting proliferation, maturation, protection, and repair of the intestinal epithelium [179]. High quantities of EGF, in particular, have been detected in HC. At an intestinal level, it inhibits cellular apoptosis, promotes the development and maturation of the intestinal barrier, and exerts a regulatory effect of intestinal inflammatory processes. EGF levels undergo a significant reduction during lactation, while higher levels are found in preterms, especially among ELBWs [131].

The heparin-binding epidermal growth factor (HB-EGF) is an EGF family glycoprotein that promotes enterocyte migration and proliferation, and also plays a protective effect against NEC. Recent studies have also shown that HB-EGF is able to regulate intestinal motility and blood flow by direct action on the enteric nervous system (ENS); this mechanism can facilitate repair processes after hypoxic damage as observed in NEC [180].

The development and maturation of intestinal functions is also promoted by specific GF, such as FGF-6, FGF-7 and IGFs, stimulating the proliferation of intestinal crypt cells and inhibiting apoptosis. Similarly, glucagon improves splancnic perfusion and promotes mucosal growth, which are effects used in the treatment of short bowel syndrome [179].

Recently, an intestinal protective role of erythropoietin has been demonstrated in terms of antiapoptotic affect and the protection of the intestinal barrier. Likewise, G-CSF is also known as hematopoietic GF, which protects the intestinal mucosa and facilitates the repairing processes after NEC [179].

Similar effects are carried out by TGF which plays a protective role against NEC since it modulates inflammatory responses and promotes mucosal barrier recovery processes. TGF is present in HC and decreases in preterm samples during lactation [181]. In addition, a negative correlation between TGF-β1 levels and BW and GA was observed, while TGF-β2 decreased in the HC of IUGR mothers with reduced food tolerance and also in HC from mothers of preterms affected by NEC [182]. 

Variations in TGF-β2 concentrations have been also related to ethnicity and maternal diet [181,183]. 

### 7.2. Neuronal Growth Factors 

Brain-derived neurotrophic factor (BDNF) and neurotropin-3 (NT-3) are growth and neurotrophic factors involved in synaptogenesis during the development and differentiation of the central and peripheral nervous system. At an intestinal level, they influence structural and functional development of ENS [184].

BDNF, the glial cell line-derived neurotrophic factor (GDNF), and NT-3 have been found in HC, showing higher concentrations in preterm samples. Another important protein, the S100B, involved in metabolic and neuronal growth, has also been detected in HC [184,185].

These recent studies confirm that HC supports and promotes the regular development of both the systemic nervous system (SNC) and ENS.

### 7.3. Angiogenic Growth Factors

Vascular endothelial growth factors (VEGF), such as platelet-derived growth factor (PDGF-BB), involved in angiogenesis and vasculogenesis mechanisms, have been found in HC, with decreasing concentrations during lactation [131,179,184,186]. These GF are probably produced by HC stem cells (SCs), and promote angiogenesis and tissue maturation. A detailed description of HM SCs is not one of the aims of this review, but can be found in the following References [187,188,189].

## 8. Pasteurization 

In NICU, HC administration is essential for nutrition and for the correct development of all organs and tissues, especially in ELBW. Since often mothers fail to supply, the use of donated HC—thoroughly subjected to the pasteurization process—is fundamental. 

There are many data about the effects of Holder Pasteurization (HP) on the nutritional properties and biological characteristics of HM, but less is known about the effects that pasteurization produces in HC. In a recent review regarding HM and pasteurization, among 44 analyzed articles, there were only six regarding HC [190,191].

The most relevant data confirmed that in HC (as it happens in mature HM), the saccharide component was not modified after pasteurization, while the immunological, immunoprotective and immunomodulating protein fractions underwent variations.

HP modifies the HC immunological profile reducing IgA, IgG and IgM; moreover, an impairment of immunoprotective activity, related to a reduction in lisozime and lactoferrin levels has also been described [190,191,192]. Innate and adaptative immunity related cytokines were not significantly altered, while among evaluated chemokines (such as IL-8, MCP-1, MIP-1β), only MIP-1β levels were reduced after HP [192]. 

Similarly, in HC, the enzymatic antioxidant activity of superoxide dismutase and glutathione peroxidase were also reduced by pasteurization. In contrast, glutathione-reductase activity and non-enzymatic properties did not show variations [193]. Only few data were available on the effects of HP on cellular GF; among the studies evaluating GM-CSF and S100B, no significant variations were shown [192,194].

To prevent significant alterations in HC biological and nutritional properties, some thermal (as high- temperature short-time) and non-thermal techniques (such as high-pressure processing, UV irradiation and ultrasonic processing) have been recently proposed as alternatives to HP [195,196,197,198].

Despite the promising effects associated with the use of these new techniques (which seem to be able to provide adequate antibacterial and antiviral safety), the few available data about nutritional, bioactive and immunological fractions conservation and the elevated costs make the clinical application of these alternative of poor reliability at the moment. Our literature review, on the small number of available data, demonstrates an open debate of HP effects on HC: further studies are needed to clarify the effects of the different pasteurization techniques on its components.

## 9. Conclusions

There is great interest in the peculiar role and the characteristics of HC in newborn nutrition. Several studies have been already performed to evaluate the features of the fluid naturally predisposed to neonatal growth and development during the very first moments of life, sustaining the delicate phase of adaptation to postnatal life, although much research is still needed to fully clarify the real benefits of such a precious liquid.

In the last few years, “omics” technologies have provided an exceptional tool to investigate the composition of many fluids and tissues, allowing a detailed description of selective gene expression, microbiota characteristics, and dynamic changes in HC proteome and metabolome compared to later stages of lactation, greatly enriching our knowledge in relation to its composition. This evidence provides the basis of our understanding the effective functions of these mediators in breastfed newborns, with their possible impact on neonatal health and maturation. HC features in preterms can be also compared with samples from mothers of full term babies to understand the possible differences between these two categories, with HC being able to modify its composition based on the necessities of each neonate. 

Moreover, in case of HC analysis, a strength point of “omics” is represented by the possibility of a non-invasive sample collection. 

The abundance of several GF in HC and their crucial role during extrauterine adaptation has also been demonstrated, with higher levels in samples from mothers of prematures, although in the future, several studies should be performed to completely understand their origins, exerted effects and the possible correlations with the interesting and fascinating population of SCs detected in HC.

Regarding the pasteurization effects on HC, there is still an open debate to evaluate the best technique to least modify its natural and beneficial components to ensure adequate quantities of donated HC those babies whose mothers are unable to provide.

Improved knowledge of different features between HC and MM, with a further comparison to FM, confirmed the arguments sustaining breastfeeding promotion and colostrum administration especially in NICU, and could also result in formulating the most suitable milk and dairy food composition to provide for each category of newborn.

## Figures and Tables

**Table 1:** Table resuming growth factors (GF) detected in human colostrum (HC), their functions and their levels in colostrum instead of mature milk (MM).

Growth Factor (GF)	Functions	Presence in Human Colostrum (HC)
Epidermal Growth Factor (EGF)	Regulation in intestinal inflammatory processes. Promotion of intestinal barrier maturation. Inhibition of intestinal epithelium apoptosis	Higher than in MM
Hepatocyte Growth Factor (HGF)	Promotion of proliferation, angiogenesis and intestinal tissue maturation via paracrine and endocrine signaling	Higher than in MM
Insulin-like Growth Factor (IGF)	Development and maturation of intestinal function. Stimulation of intestinal crypt cells and inhibition of apoptosis	Higher than in MM
Transforming Growth Factor (TGF)	Modulation of inflammation. Promotion of mucosal reparation. Protection against NEC damage, promoting epithelium reparation	Higher than in MM
Fibroblast Growth Factor (FGF-6, FGF-7)	Development and maturation of intestinal function	Higher than in MM
Granulocyte-colony stimulating Factor (G-CSF)	Hematopoietic growth factor, protection of intestinal mucosa. Promotion of mucosal repair after NEC	Higher than in MM
Heparin-binding Epidermal Growth Factor (HB-EGF)	Promotion enterocytes migration and proliferation. Protection against NEC. Action on ENS. Reparation of intestinal epithelium after hypoxia	Higher than in MM
TGF-B1	Protection of intestinal mucosa	Negative correlation with BW and GA
TGF-B2	Promotion of gut maturation. Suppression of macrophage inflammatory responses in the developing intestine. Protection against inflammatory mucosal injury	Reduced in HC of IUGR with feeding intolerance or NEC
Brain Derived Neurotrophic Factor (BDNF) and Neurotropin NT-3	Neuronal GF involved in synaptogenesis. Promotion of enteric nervous system development	High levels in HC
Glial-cell line derived neurotrophic Factor (GDNF)	Development and survival of the enteric neurons, promotion of survival and morphological differentiation of dopaminergic neurons	High levels in HC
S100B	Promotion of neurogenesis, brain repair/regeneration	High levels in HC
Platelet-derived Growth Factor	Promotion of angiogenesis, vasculogenesis and tissue maturation	High levels in HC

NEC = Necrotizing enterocolitis; ENS = Enteric nervous system; BW = Birth weight; GA = Gestational age; IUGR = intrauterine growth restriction; HC = Human Colostrum; MM = Mature Milk.
